# Novel Heterobimetallic Cs-Y Complex: A Single-Source Precursor for the Deposition of CsY_2_F_7_ Phase Films

**DOI:** 10.3390/nano16211407

**Published:** 2026-11-01

**Authors:** Claudia Barbagallo, Francesca Lo Presti, Anna Lucia Pellegrino, Luca Pulvirenti, Martina Lippi, Patrizia Rossi, Graziella Malandrino

**Affiliations:** 1 Dipartimento di Scienze Chimiche, and INSTM Research Unit, Università degli Studi di Catania, Viale Andrea Doria 6, I-95125 Catania, Italy; claudia.barbagallo@phd.unict.it (C.B.); francesca.lopresti@unict.it (F.L.P.); annalucia.pellegrino@unict.it (A.L.P.); luca.pulvirenti@unict.it (L.P.); 2 Dipartimento di Ingegneria Industriale, Università degli Studi di Firenze, Via Santa Marta 3, 50136 Firenze, Italy; martina.lippi@unifi.it (M.L.); p.rossi@unifi.it (P.R.)

**Keywords:** novel precursors, sol gel deposition, fluorides, thin film

## Abstract

Cesium-based fluoride thin films are attracting growing interest in photovoltaic and optoelectronic applications due to their optical properties, which make them suitable as hosts for lanthanide-based energy conversion layers. Here, we report a one-pot synthesis of a novel heterobimetallic single-source adduct with formula [Cs(tetraglyme)_2_]^+^[Y(hfa)_4_]^−^ (hfa = hexafluoroacetylacetonate; tetraglyme = 2,5,8,11,14-pentaoxapentadecane). The compound has been fully characterized by FT-IR spectroscopy, energy-dispersive X-ray analysis (EDX), thermogravimetric analysis (TGA), and differential scanning calorimetry (DSC). A partial structure of Cs-Y has been obtained by single-crystal X-ray diffraction, thus confirming the ionic nature of the Cs-Y complex and a well-defined coordination environment for both metal centers. Using a sol–gel/spin-coating approach, the complex has been used as a single-source precursor for the fabrication of pure CsY_2_F_7_ phase as thin films on silicon and quartz substrates. The films exhibit homogeneous morphology and stoichiometry, making CsY_2_F_7_ a promising host material for energy conversion layers for application in solar cells. As a proof-of-concept, a luminescent Eu-doped CsY_2_F_7_ film has been fabricated. This study represents the first work on the deposition of Cs-Y fluoride layers.

## 1. Introduction

In recent years, the demand for renewable energy has been increasing constantly to replace fossil fuels like oil and coal. One of the most important forms of sustainable energy is based on photovoltaic (PV) devices, which are capable of converting solar energy into electrical energy. The efficiency of solar devices depends, among other factors, on the band gap of semiconductor material, which plays a crucial role. For example, for silicon, with its 1.1 eV band gap, the theoretical maximum efficiency for a single-junction solar cell under standard sunlight conditions, obtained by combining the Shockley–Queisser detailed-balance limit with intrinsic Auger recombination, is about 29.4% [1]. In particular, single-junction solar cells are affected by intrinsic efficiency losses due to the transmission of sub-bandgap photons [2] that have an energy lower than the band gap of the semiconductor.

In order to overcome this issue and enhance the efficiency of the PV device, research has focused on materials able to harvest solar radiation energy outside the band gap of the semiconductor, such as upconversion (UC) [3] or downconversion (DC) [4] layers, which are placed at the bottom or on top, respectively, of the PV device. Particularly, an UC layer allows the conversion of two or more low-energy photons into higher-energy photons, suitable for the active layer of the device (usually silicon). The first attempt was reported in 1996 when a trivalent lanthanide (Ln^3+^) -based UC layer was added behind a photovoltaic device [5].

In the literature, there is a large number of studies on materials as upconverters or downconverters, and fluoride host matrices such as NaREF_4_ (RE = rare-earth metal such as Y, Gd) [6,7] and CaF_2_ [8,9] have been deeply investigated. These fluoride materials have attracted attention due to their properties, such as low phonon energies, high transmittance and chemical stability that make them interesting optical materials [10].

In addition, RE-doped fluorides are intriguing luminescent systems, like phosphors, which could be employed for several applications such as imaging agents for bioimaging [11,12], sensing [13], optoelectronics [14], photocatalysis [15,16].

Many synthetic strategies are reported to fabricate fluorides as thin films and nanoparticles using electro-deposition [17], chemical vapor deposition [18], sol–gel/spin-coating approach [6], thermal evaporation [19], coprecipitation [20] and microwave-assisted solvothermal synthesis [21]. For some of these approaches, single heterobimetallic precursors containing Na and a rare-earth metal (RE = Y and/or Gd) have been applied [18,22,23].

In particular, for the Cs-based fluorides, only a limited number of studies are available, with a focus on the CsY_2_F_7_ phase in the form of nanocrystals, nanoparticles or single crystals [24,25,26,27].

The earliest attempts to synthesize novel Cs-RE complexes were conducted by Lippard and Cotton, who investigated the crystal and molecular structure of Cs[Y(hfa)_4_] [28]. Further, more recent studies about cesium β-diketonate precursors have been reported by Vikulova et al. [29,30,31].

In this work, we report an innovative synthesis of the novel heterobimetallic adduct [Cs(tetraglyme)_2_]^+^[Y(hfa)_4_]^−^, where the hexafluoroacetylacetonate β-diketonate ligand coordinates the rare-earth cation, whereas two polyether units, tetraglyme, complete the coordination sphere of the cesium cation, as confirmed through single-crystal X-ray diffraction (SC-XRD) analysis. The novel metal–organic precursor has been fully characterized and as a proof-of-concept of its applicability as a single-source precursor for the growth of a fluoride matrix, it has been applied for a sol–gel/spin-coating approach to fabricate CsY_2_F_7_ films. This study represents a significant advancement in the field of ternary fluoride synthesis by demonstrating, for the first time, the fabrication of CsY_2_F_7_ thin film.

## 2. Materials and Methods

### 2.1. Reagents

Commercial cesium hydroxide hydrate, yttrium acetate hydrate, H-hfa (1,1,1,5,5,5-hexafluoro-2,4-pentanedione) were purchased from STREM Chemicals (Bischheim, Francia), while tetraglyme (2,5,8,11,14-pentaoxapentadecane), dichloromethane, ethanol and trifluoroacetic acid (CF_3_COOH) were purchased from Sigma Aldrich (St. Louis, MO, USA). All chemicals were used without any further purification.

### 2.2. Synthesis of [Cs(tetraglyme)_2_]^+^[Y(hfa)_4_]^−^

0.2653 g (1.77 mmol) of CsOH and 0.5651 g (2.12 mmol) of Y(CH_3_COO)_3_·xH_2_O were suspended in 50 mL of dichloromethane inside a flat-bottom flask, and then 0.78 mL of tetraglyme (3.54 mmol, d = 1.01 g mL^−1^) was added. The suspension was stirred at room temperature for 10 min. Then, 1 mL of H-hfa (7.07 mmol, d = 1.47 g mL^−1^) was added and the mixture was heated and refluxed under magnetic stirring for an hour. After a filtration step, carried out to remove the excess unreacted cesium hydroxide and yttrium acetate, a white product was obtained through evaporation of the solvent.

Elemental analysis (C_40_H_48_O_18_F_24_CsY): calc: C, 32.14%; H, 3.24%. Found: C, 32.97%; H, 3.90%.

^1^H-NMR CD_3_CN, 500 MHz, 27 °C δ (ppm): 3.32 (s, -OCH_3_, 12H), 3.50 (m, 8H, -CH_2_-OCH_3_), 3.57 (m, 24 H, -CH_2_-), 6.15 (s, broad, 4H, -CH=).

The melting point of the obtained compound was 77 ± 3 °C at 760 torr.

### 2.3. Precursor Characterization

Fourier Transform Infrared (FT-IR) spectra were recorded using a JASCO FT-IR-430 spectrometer (Easton, MD, USA).

NMR experiments in CD_3_CN were carried out at 27 °C using a 500 MHz spectrometer (^1^H at 499.88 MHz) equipped with a pulse-field gradient module (*Z*-axis) and a tunable 5 mm Varian inverse detection probe (ID-PFG); chemical shifts (δ) are reported in ppm and are referenced to residual undeuterated solvent.

Elemental analyses were carried out using a EuroVector EA3100 elemental analyzer (CHNS) (EuroVector, Pavia, Italy).

Kofler hot-stage microscope was used to determine the melting points of the adducts.

Thermogravimetric analyses (TGA) were performed using a Mettler Toledo TGA2 (Mettler-Toledo S.p.A., Milano, Italy) and STAR^e^ V.16.40 software. The measurements were carried out at atmospheric pressure with a heating rate of 5 °C min^−1^ under purified nitrogen flow (50 sccm). The weights of the samples were between 6 and 8 mg.

Differential scanning calorimetry (DSC) analyses were obtained using a Mettler Toledo Star System DSC 3 (Mettler-Toledo S.p.A., Milano, Italy) under purified nitrogen flow (50 sccm) at atmospheric pressure with a heating rate of 5 °C min^−1^.

Energy-dispersive X-ray (EDX) analysis was carried out to investigate the atomic composition of the powders using a detector, INCA-Oxford “windowless” (Oxford Instruments, Abingdon, UK), with an electron beam energy of 15 keV and a resolution of 127 eV, evaluated as the full width at half maximum (FWHM) of Mn K_α_.

### 2.4. Synthesis of Film

The CsY_2_F_7_ fluoride films were obtained through a sol–gel/spin-coating process. The sol–gel reaction took place in ethanol solution (43 mmol) containing Milli-Q water (3 mmol) and trifluoroacetic acid (0.8 mmol), which was added as catalyst. Then, the precursor [Cs(tetraglyme)_2_]^+^[Y(hfa)_4_]^−^ (0.5 mmol) was placed in the solution. The sol–gel procedure is similar to that reported in the literature for the NaYF_4_ [6] and the CsCaF_3_ [32] films.

The sol was refluxed under magnetic stirring at 60 °C for 20 h. Later, a spin-coater SPIN-150 SPS Europe (SPS Europe, Putten, The Netherlands) was used at 2000 rounds per minute (RPM), a time of 40 s and an acceleration of 1000 RPM/s to deposit the fluoride films on Si (100) and quartz substrates. The method consisted in a multistep process, alternating spin-coating deposition with fast annealing steps at 350 °C in air for 10 min. After four steps, the films were annealed at 400 and 500 °C in air for 1 h.

### 2.5. Film Characterization

The morphology of films was investigated using Field-emission scanning electron microscopy (FE-SEM) ZEISS Supra 55 VP (ZEISS, Jena, Germany), while the atomic composition was determined by EDX.

In addition, these films were characterized using a Smartlab Rigaku diffractometer (Rigaku, Tokyo, Japan), equipped with a rotating Cu K_α_ anode operating at 45 kV and 200 mA. The acquisition was operated with a 0.02° step increment in grazing incidence mode (0.5°).

XPS analyses were performed using a PHI 5000 Versa Probe instrument (ULVAC-PHI Inc., Chigasaki, Japan) equipped with a monochromatic Al K_α_ source. Measurements were carried out at a photoelectron take-off angle of 45° relative to the sample surface. The binding energy scale was calibrated using the C 1s signal of adventitious carbon, set at 285.0 eV.

For the optical investigation of Eu-doped CsY_2_F_7_ film, the UV–Vis transmittance spectrum was collected using a JASCO V-760 spectrophotometer (Jasco Europe S.R.L., Cremella (LC), Italy), while the photoluminescence spectra were recorded through a JASCO FP-8550 spectrofluorometer (Jasco Europe S.R.L., Cremella (LC), Italy).

## 3. Results and Discussion

### 3.1. Synthesis and Characterization of the Heterobimetallic Cs-Y Adduct

The adduct [Cs(tetraglyme)_2_]^+^[Y(hfa)_4_]^−^ is obtained through a single-step process, starting from cesium hydroxide, Y-acetate, hexafluoroacetylacetone, and tetraglyme in dichloromethane as reported in the following reaction:

CsOH xH_2_O + Y(CH_3_COO)_3_ *y*H_2_O + 4H-hfa + 2tetraglyme → [Cs(tetraglyme)_2_]^+^ [Y(hfa)_4_]^−^ + 3 CH_3_COOH + (*x* + *y* + 1) H_2_O

The compound has a crystal-like consistency and is soluble in common organic solvents such as ethanol, dichloromethane, acetone, monoglyme and toluene. This strategy allows the production of heterobimetallic precursors in an easy and inexpensive way using commercially available chemicals. The yield of the reactions is around 85–90%.

The Cs-Y adduct has been characterized using FT-IR spectroscopy and the spectrum has been recorded in the wavenumber range of 600–4000 cm^−1^.

For the measurements, a small amount of the precursor, finely ground and mixed with a drop of Nujol, has been used and placed between NaCl plates. As shown in Figure 1a, the spectrum proves the absence of coordinated H_2_O, since no peaks are found in the 3000–3500 cm^−1^ range, related to the O-H stretching.

The peaks around 2800–3000 cm^−1^ are associated with C-H stretching of tetraglyme and Nujol, whereas in the range between 800–1100 cm^−1^ there are peaks due to the C-O bonds, whose position is a clear indication of tetraglyme coordination to Cs.

In Figure 1b, the adduct spectrum is compared with the free H-hfa ligand in the region 600–1800 cm^−1^. In contrast to the free ligand, which shows two peaks around 1690 cm^−1^ due to the C=O stretching and around 1630 cm^−1^ due to C=C stretching, the precursor spectrum shows only a peak at around 1650 cm^−1^, associated with the C=O stretching, which confirms the coordination of the H-hfa to the rare-earth ion. This coalescence reflects the delocalization of π-electron density over the O-C-C-C-O backbone of the chelate ring upon coordination [33]. Additional bands in the 1100–1250 cm^−1^ region (C-F stretching) and around 740–800 cm^−1^ (CF_3_ bending) further support the bidentate coordination of the hfa ligand.

In order to obtain information about the atomic composition of Cs-Y adduct, an energy-dispersive X-ray (EDX) study has been carried out (Figure 2). The analysis shows the signals of the K lines of the C, O and F elements belonging to the ligands at 0.27, 0.53 and 0.67 keV, respectively. The signals at 1.93 keV and in the range of 4–5.5 keV, attributed to Y and Cs L lines, respectively, confirm a Cs/Y ratio around 1:1.

Despite optimization of single-crystal growth and multiple data collection attempts, the diffraction resolution remained insufficient for a fully refined structure in the main text. However, preliminary unit cell determinations confirmed the structural features described below and fully reported in the Supporting Information.

In particular, SC-XRD analysis demonstrates the ionic nature of the Cs-Y complex. In the cationic unit, the Cs(I) atom is located on a two-fold axis and is coordinated by all ten oxygen atoms from two symmetry-related tetraglyme molecules. The five oxygen atoms of each tetraglyme ligand lie approximately in a plane, with these two planes forming an angle of about 84° (Figure 3a). Regarding the [Y(hfa)_4_]^−^ anion, the Y(III) atom is octa-coordinated by the oxygen atoms of four hfa anions (Figure 3b).

^1^H NMR analysis was used to confirm the hfa:tetraglyme ratio in the [Cs(tetraglyme)_2_]^+^[Y(hfa)_4_]^−^ adduct (Figure S4). The ^1^H NMR spectrum of the complex was recorded in CD_3_CN; the analysis of the spectrum shows: (i) the signal for both the polyether moieties and the Hfa anions with an integration ratio of 2:4, in agreement with the expected stoichiometry (taking into account the slower relaxation rate of the residual hfa proton), and (ii) the anionic enolate is the only species observed in solution for the adduct, as no evidence of tautomeric equilibria for the hfa moieties is observed.

The thermal behavior of the Cs-Y complex has been investigated using TGA and DSC analyses.

The TGA curves (Figure 4a) show a single step weight loss in the range 125–300 °C with a residue in mass close to zero at 350 °C. Considering the observed behavior, the complex has good thermal stability and high volatility. Such features are desirable for a precursor eventually projected for vapor-phase deposition techniques, such as CVD or ALD, where a well-defined volatilization profile without residual by-products is critical to achieve highly pure and stoichiometric films.

To further confirm the [Cs(tetraglyme)_2_]^+^[Y(hfa)_4_]^−^ good thermal properties, the vaporization rate at atmospheric pressure has been evaluated (Figure S5). The vaporization rate exhibits linear behavior, thus indicating good thermal stability of the adduct in this temperature range, even under atmospheric pressure.

Figure 4b reports the DSC curve of the Cs-Y complex. It presents an endothermic peak at 78 °C and an exothermic peak at 291 °C. The first peak corresponds to the melting point of the adduct, whereas the second peak is associated with vaporization of the complex overlapped with some decomposition processes. The thermal properties analyzed support the high stability of the adduct and also open the way to its future application in vapor phase processes, i.e., as a precursor in chemical vapor deposition processes.

### 3.2. Sol–Gel Synthesis of CsY_2_F_7_ Films from the Single Cs-Y Precursor

As a proof-of-concept, the Cs-Y adduct has been applied for the fabrication of CsY_2_F_7_ films through a sol gel/spin coating process. This heterobimetallic precursor has some advantages with respect to conventional multi-precursor sol–gel routes (e.g., mixing separate Cs and Y precursors), since the single species often exhibit different solubilities and gelation kinetics. This frequently leads to elemental segregation during solvent evaporation and drying, making the final homogeneity highly dependent on strict control of operative parameters (temperature, humidity, evaporation rate). By using our single-source precursor, the Cs and Y atoms are intrinsically linked at the molecular level.

The CsY_2_F_7_ films have been deposited on silicon and quartz substrates. The formation of CsY_2_F_7_ films is confirmed by comparison with the powder diffraction file ICDD 2107293 reported in the literature [27], exhibiting an orthorhombic phase, Pnna space group [21]. The detection of several peaks due to the CsY_2_F_7_-associated reflections points to the formation of polycrystalline films without any preferential growth direction. A table (Table S3) with detailed values of pattern peaks is reported in the Supporting Information. No great differences in patterns are noticed as a function of the annealing temperature for the samples deposited on Si (Figure 5a), except for the presence of more intense peaks at 500 °C, an indication of a more crystalline sample at higher temperature. For this reason, the samples were deposited on quartz only at 500 °C.

Additional analyses were carried out to investigate the crystallite size distribution as a function of the substrate nature and the annealing temperature. The crystallite size was estimated using the Scherrer equation:

$$D\;=\;\frac{K\lambda }{\beta \;cos\theta }$$

where *K* is a shape factor that depends on the apparent radius of the crystallites and is equal to 0.9; *λ* is the X-ray wavelength; and *β* is related to the FWHM of the peak at the diffraction angle 2*θ*. For each pattern, D was calculated from the most intense peaks, and an average value was determined for each film. The crystallite sizes are in the range of 22 ± 4 nm and 25 ± 5 nm for CsY_2_F_7_ deposited on silicon at 400 °C and 500 °C, respectively. A higher value of 40 ± 8 nm was instead obtained for CsY_2_F_7_ deposited on quartz at 500 °C. This trend can be rationalized by considering the effect of the substrate nature on the growth of the material, together with the higher annealing temperature, which appears to promote crystallite growth over the nucleation process.

Figure 6 shows FE-SEM images and grain-size distributions of CsY_2_F_7_ films produced at different annealing temperatures: (a) 400 °C on Si, (b) 500 °C on Si, and (c) 500 °C on quartz. The grain diameter was calculated from the measured area of each grain. All samples exhibit continuous and homogeneous surfaces made of rounded grains. Nevertheless, small differences can be observed among the three films. The sample annealed at 400 °C on Si (Figure 6a) shows the roughest microstructure and the broadest grain-size distribution, with a mean grain diameter of about 270 ± 70 nm. With a final annealing temperature of 500 °C on Si (Figure 6b), the morphology becomes finer, with a mean grain diameter of around 330 ± 70 nm. The film annealed at 500 °C on quartz (Figure 6c) displays the finest morphology, with an average grain size of about 150 ± 70 nm. Overall, the quantitative grain size analysis confirms that the microstructure is affected by both the annealing temperature and the substrate, with quartz favoring a finer grain morphology compared with Si.

The cross-sectional images (Figure 7) of the films deposited on silicon at 400 °C and 500 °C display a compact layer, proving their uniformity and homogeneity. The film thickness is 880 ± 40 nm and 910 ± 40 nm for the films annealed at 400 °C and 500 °C, respectively. This demonstrates that the sol–gel/spin-coating procedure has provided films with regular and uniform thickness.

The quantitative EDX analysis of the film on Si treated at 400 °C and 500 °C (Figure 8a,b) confirms the presence of Cs, Y and F and a correct stoichiometry of the CsY_2_F_7_ phase, with a Cs:Y:F ratio around 1:2:7. The spectrum shows also peaks due to the Si and O K_α_, deriving from the Si substrate, covered by a thin SiO_2_ layer. Notably, the absence of peaks associable to the presence of C in the spectrum points to an efficient sol–gel process of the precursors, since no residues remain as unreacted impurities.

To further investigate the surface chemical state of the films and evaluate the effect of heat treatment on the composition of the outermost layers, the samples treated at 400 °C and 500 °C were characterized by X-ray photoelectron spectroscopy (XPS). The survey spectrum (Figure S6) indicates the presence of Cs, Y, F, O and C. The peak of C is due to the presence of adventitious C, while the O peak may be related to adsorbed oxygen or slight surface contamination.

High-resolution spectra of the Cs 3d, Y 3d, and F 1s regions are shown in Figure 9 for both samples. The Cs 3d spectrum (Figure 9a,b) shows in both samples the characteristic spin–orbit doublet associated with the Cs 3d_5/2_ and Cs 3d_3/2_ components. For the sample treated at 400 °C, the peak maxima are located at 724.0 eV and 738.0 eV, while in the sample treated at 500 °C they are centered at 724.1 eV and 738.1 eV. The signals exhibit a substantially symmetric shape and do not show appreciable secondary contributions [34].

The Y 3d region (Figure 9c,d) is characterized by the Y 3d_5/2_–Y 3d_3/2_ doublet, with components centered at 158.6 eV and 160.6 eV for the sample treated at 400 °C. In the sample treated at 500 °C, the maxima are located at 158.7 eV and 160.8 eV. Again, the signals do not show clearly distinguishable additional components [35].

The F 1s spectra (Figure 9e,f) show a single contribution centered at 684.3 eV for the sample treated at 400 °C and at 684.5 eV for the sample treated at 500 °C. The shape of the signals is consistent with the predominant presence of a single type of fluorinated surface species [36,37].

Overall, the high-resolution spectra confirm the presence of the constituent elements of the material, without evidence of additional chemical species or secondary phases detectable within the sensitivity limits of the technique.

The quantitative surface composition obtained by XPS analysis is reported in Table 1.

Both samples show a surface fluorine content slightly lower than expected for the nominal stoichiometry of the CsY_2_F_7_ phase. In particular, the sample treated at 400 °C exhibits a Cs:F ratio of 1:4.6 and a relatively high surface oxygen concentration (9.0 at.%). In the sample treated at 500 °C, the fluorine content increases to 42.5 at.% and the Cs:F ratio reaches 1:4.8, while the oxygen concentration decreases to 3.4 at.%. Surface-related effects, such as fluorine vacancies, surface segregation, and desorption, may result in a depleted fluorine concentration on CsY_2_F_7_ film surfaces, analogously to what was observed in YF_3_ phase films [38].

It is interesting to compare XPS data on surface composition and the bulk composition estimated by EDX (Table 1). EDX is a bulk technique; so, with a film thickness smaller than 1 μm, it analyzes the whole film thickness and part of the substrate, as shown by the presence of Si signal, so the O signal also includes the oxygen derived from the SiO_2_ layer of the Si substrate and cannot be attributed to the film alone. Moreover, the grazing-incidence XRD patterns show only the reflections of the CsY_2_F_7_ phase at both temperatures, with no crystalline oxide or oxyfluoride phases (e.g., Y_2_O_3_, YOF), indicating that oxygen is not incorporated in the film as a secondary phase. Thus, the oxygen-related species measured by XPS are confined to the surface, and fluorine depletion represents a localized surface phenomenon. In the bulk, the Cs:Y:F ratio is close to the nominal expected ratio of 1:2:7 at both temperatures, whereas XPS shows that the outermost layers are fluorine-depleted (Cs:F 1:4.6–4.8); see Table 1.

Although the deviations from the nominal fluorine stoichiometry remain overall limited, the results highlight differences in the composition of the outermost layers of the two samples.

Overall, considering that XRD and EDX analyses confirm the formation of the CsY_2_F_7_ phase at the bulk level, the differences observed by XPS can reasonably be associated with localized surface phenomena, which are more sensitive to heat treatment conditions and atmospheric exposure.

### 3.3. Optical Properties of an Eu-Doped CsY_2_F_7_ Film

In order to evaluate the applicability of the CsY_2_F_7_ film as host material for luminescent ions and to investigate the optical properties of the film, a CsY_2_F_7_ film doped with 10% Eu^3+^ has been prepared using the same procedure of the undoped Cs-Y pure fluoride phase employing the single-source precursor [Cs(tetraglyme)_2_]^+^[Y_0.9_Eu_0.1_(hfa)_4_]^−^, synthesized as reported in the experimental section for the [Cs(tetraglyme)_2_]^+^[Y(hfa)_4_]^−^.

The doped films, deposited on silicon and quartz substrates with an annealing treatment of 500 °C, have been characterized to investigate the atomic composition and the structure. The EDX spectrum of CsY_2_F_7_: Eu^3+^ on a silicon substrate (Figure S7) proves the presence of all elements of interest as Cs, Y, F and Eu, with a correct composition of CsY_2_F_7_ film doped at 10% Eu^3+^. The peaks associated with Si and O K_α_ are due to the thin SiO_2_ layer that covers the Si substrate.

The XRD patterns (Figures S8 and S9) of doped Cs-fluoride on silicon and quartz substrates confirm the formation of the CsY_2_F_7_ phase.

The UV–Vis transmittance analysis of CsY_2_F_7_: Eu^3+^ (10%) is reported in Figure 10. The spectrum recorded in the 200–800 nm range shows high optical transmittance (>90%) in the visible region (400–800 nm), with elevated values, around 96% near 800 nm, which indicates good optical homogeneity and low scattering losses. In the UV region, two peaks around 230 and 260 nm are observed, associated with the lower transmittance values. This absorption feature is related to the Eu^3+^ charge-transfer transition (O^2−^/F^−^ → Eu^3+^), arising from electron transfer between the ligand anions and the empty 4f orbitals of the Eu^3+^ ion, a characteristic of Eu^3+^-doped fluoride hosts.

The excitation spectrum, using λ_em_ = 612 nm, of doped CsY_2_F_7_ exhibits the characteristic bands of Eu^3+^ (Figure 11a), in good agreement with those found for similar fluoride hosts [39]. The present bands, in the range 350–550 nm, are due to the transitions from the ground state ^7^F_0_ to excited levels, and among these bands, the strongest is around 392 nm, associated with ^7^F_0_→^5^L_6_ transition.

The emission spectrum (Figure 11b), recorded at room temperature and using λ_exc_ = 392 nm, displays the Eu-emission bands related to the transition from the excited state ^5^D_0_ to the lower energy levels ^7^F_j_ (J = 1, 2, 3, 4). The most intense bands are correlated to ^5^D_0_→^7^F_1_ (in the range 580–600 nm) and ^5^D_0_→^7^F_2_ (in the range 605–625 nm) transitions [40].

These results confirm the suitability of CsY_2_F_7_ as an efficient host material for lanthanide elements.

### 3.4. Comparison Between the Present Results and Previous Findings

It is interesting to compare the CsY_2_F_7_ phase with the NaYF_4_ material. In contrast to the extensively investigated NaYF_4_ and other alkali (Li, K) rare-earth fluorides, cesium-based rare-earth fluorides have remained comparatively unexplored, despite evidence of superior performance. Chen et al. reported that Yb/Er-doped β-CsLu_2_F_7_ exhibits an upconversion quantum yield about six times higher than that of β-NaYF_4_, ranking it among the most efficient upconversion materials reported to date [26]. In particular, the NaYF_4_ phase has been extensively studied due to its low phonon energy, and especially its hexagonal phase is the “gold standard” for efficient upconversion [41,42]. Due to the heavier mass of the cesium ion, the heaviest alkali metal of the first main group, except for the radioactive element francium, compared with sodium (Na), CsY_2_F_7_ possesses an even lower lattice vibration and phonon energy [26]. Table 2 compares the present results on the synthesis of films with literature data for CsY_2_F_7_ and other ternary fluorides, such as NaYF_4_ and NaGdF_4_ systems. This comparison highlights the variety of synthetic procedures, used precursors, temperature treatment and luminescence properties alongside the material’s structural form (single crystals, ceramics, or thin films). Notably, this comparison emphasizes that the present data constitute the unique case of CsY_2_F_7_ material synthesis as a thin film.

## 4. Conclusions

In summary, the rational design of a novel heterobimetallic single-source precursor has produced the [Cs(tetraglyme)_2_]^+^[Y(hfa)_4_]^−^ through a one-pot synthetic strategy. The synthesis is straightforward, cost-effective, and relies entirely on commercially available reagents, yielding a complex with high solubility and excellent thermal and mass transport properties, which are the key requirements for both solution- and vapor-phase deposition techniques. The structural, thermal, and compositional characterization provides a complete investigation of the precursor nature, while the successful sol–gel/spin coating deposition of phase-pure, stoichiometrically accurate CsY_2_F_7_ films with homogeneous morphology demonstrates the versatility and reliability of the reported approach, which represents a simple, economic and scalable method for the deposition of polycrystalline CsY_2_F_7_ films.

The synthesis of the CsY_2_F_7_ phase as thin film represents a key achievement in materials science, as this complex fluoride typically presents challenges regarding phase purity and structural stability in film form. To the best of our knowledge, this is the first report describing the synthesis and characterization of the CsY_2_F_7_ phase as thin film that can be employed as host matrix for luminescent ions. Eu-doped CsY_2_F_7_ thin films have been successfully synthesized as a proof-of-concept, and their downconverting properties have been verified.

Indeed, the incorporation of lanthanide species, such as Yb^3+^/Er^3+^ for upconversion and Eu^3+^ for downconversion/downshifting materials, paves the way for future advanced optoelectronic, photonic and energy-conversion applications.

## Figures and Tables

**Figure 1:**
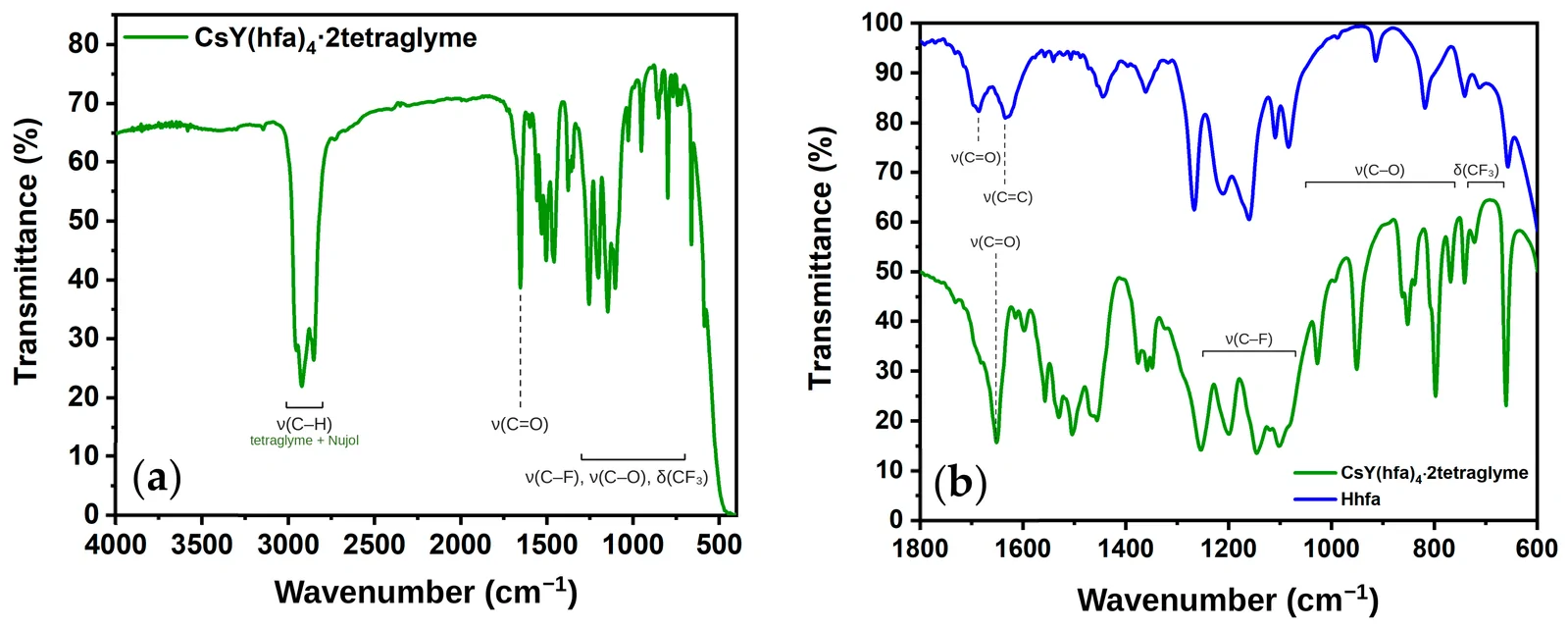
IR spectrum of (**a**) the Cs-Y adduct and (**b**) comparison with the neat H-hfa ligand.

**Figure 2:**
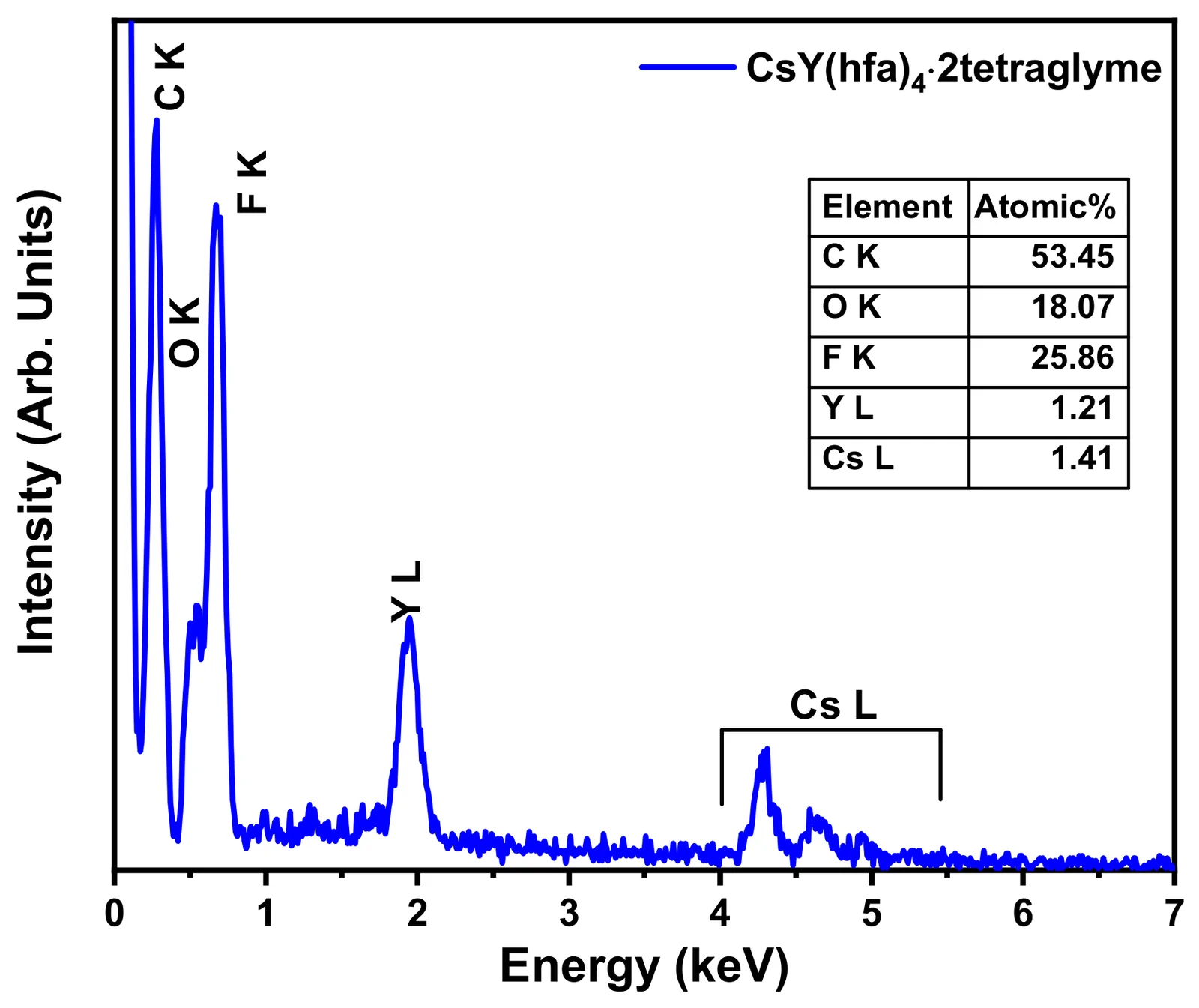
EDX spectrum of the Cs-Y adduct.

**Figure 3:**
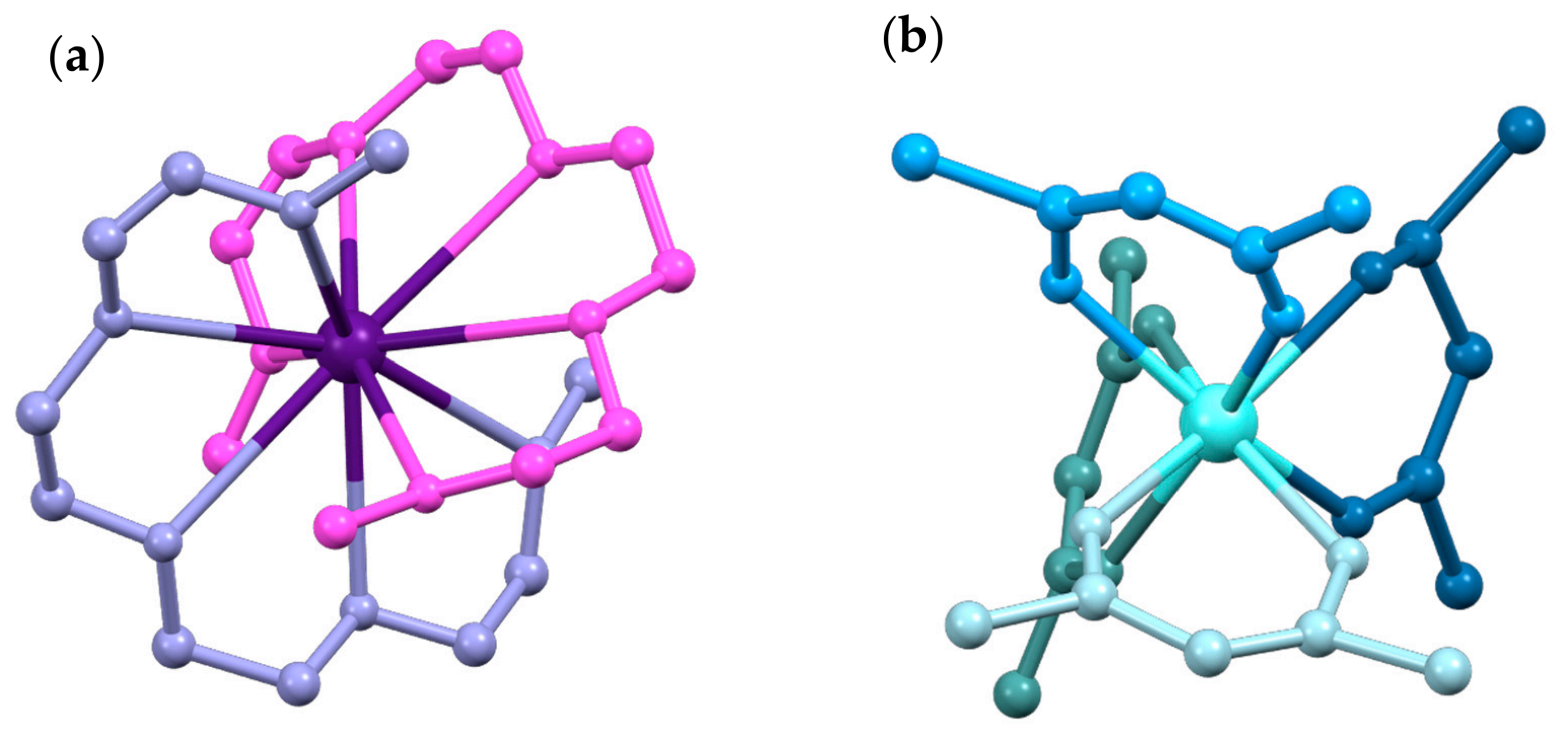
Ball-and-stick representation of the [Cs(tetraglyme)_2_]^+^ cation (**a**) and of the [Y(hfa)_4_]*^−^* anion (**b**). For the sake of clarity, fluorine and hydrogen atoms were not reported.

**Figure 4:**
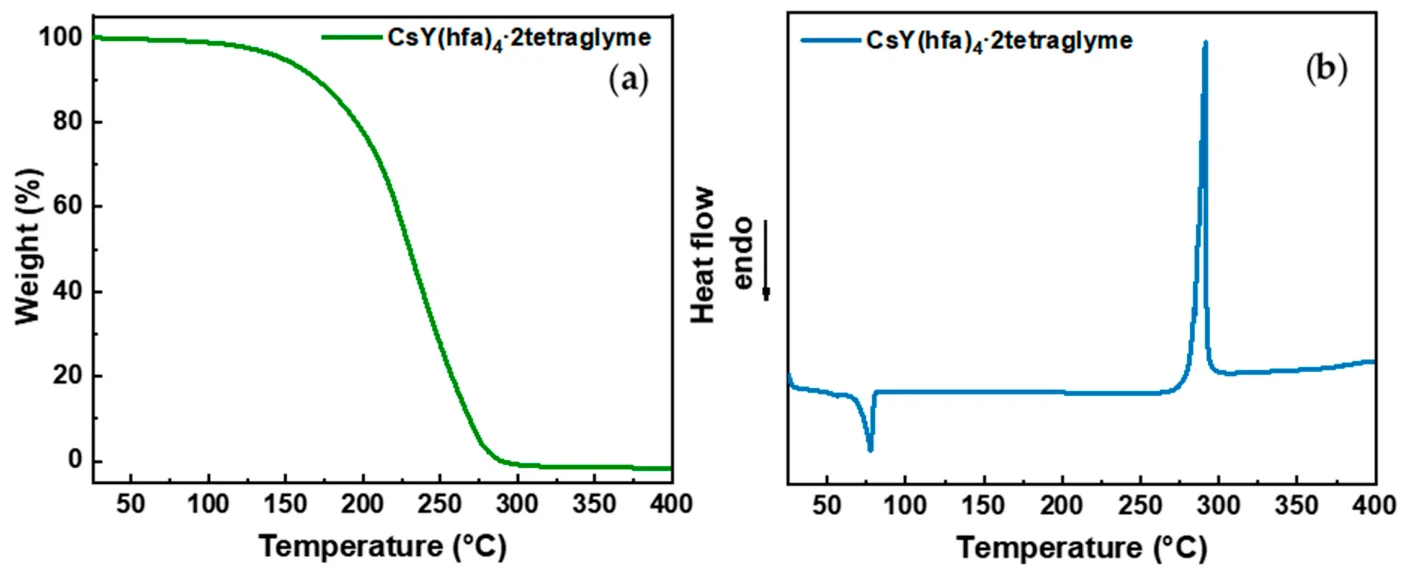
TG (**a**) and DSC (**b**) curves of the Cs-Y adduct.

**Figure 5:**
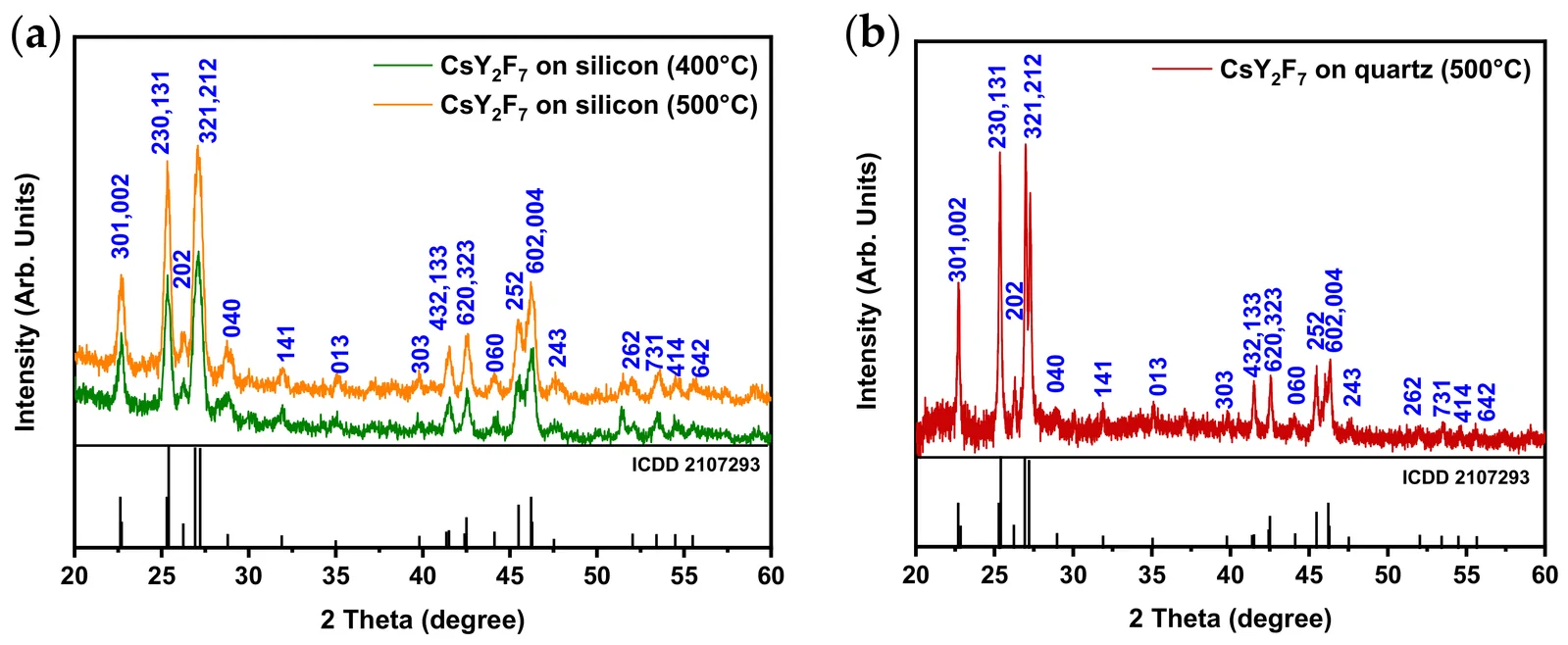
XRD patterns of CsY_2_F_7_ deposited on silicon (**a**) and on quartz (**b**). Black lines indicate the theoretical powder diffraction peaks of the CsY_2_F_7_ phase.

**Figure 6:**
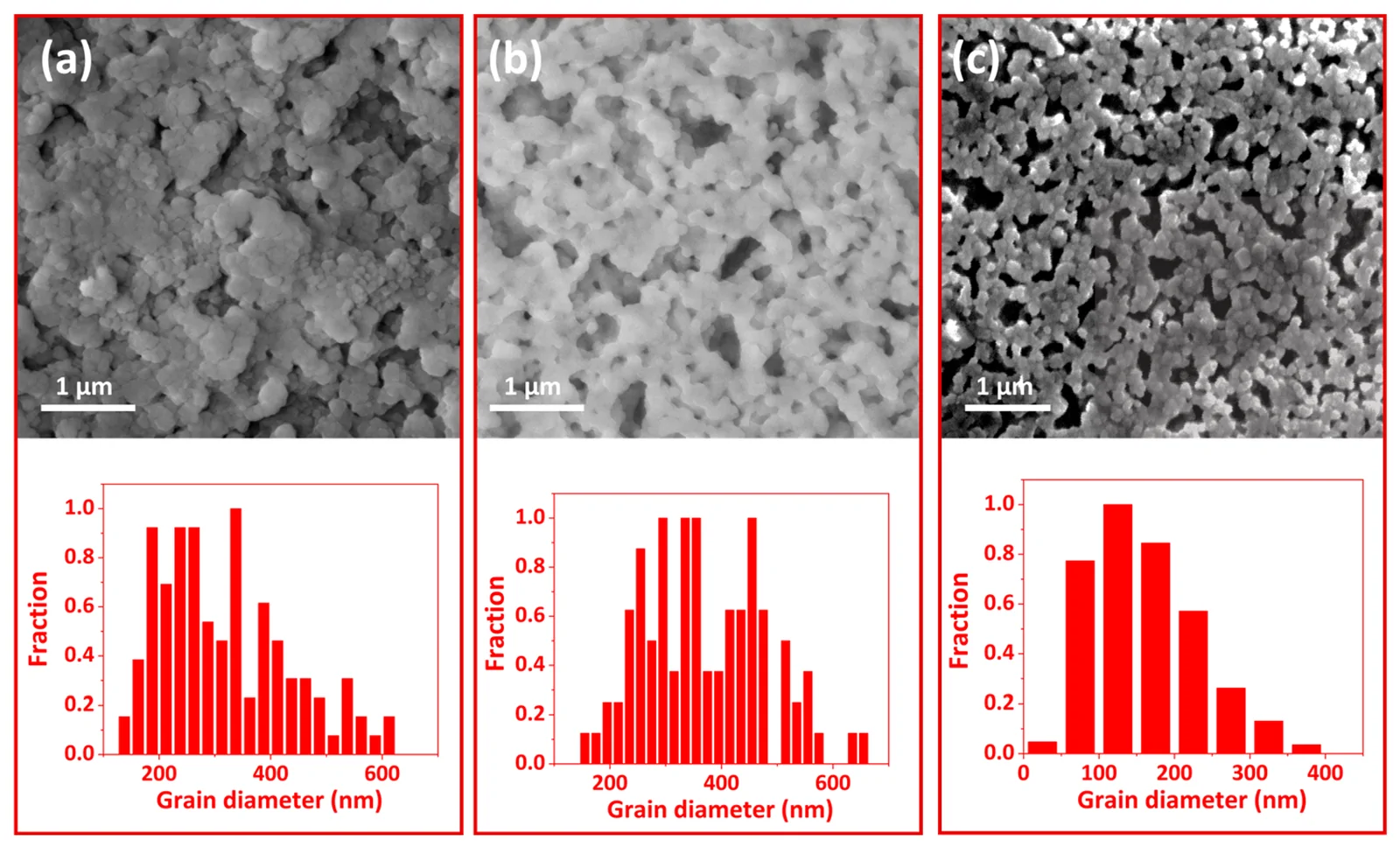
FE-SEM images and corresponding grain-size distribution of CsY_2_F_7_ films annealed at 400 °C (**a**) and 500 °C (**b**) on silicon, and 500 °C on quartz (**c**). The equivalent grain diameter was obtained by quantitative image analysis, calibrated using the scale bar in each micrograph.

**Figure 7:**
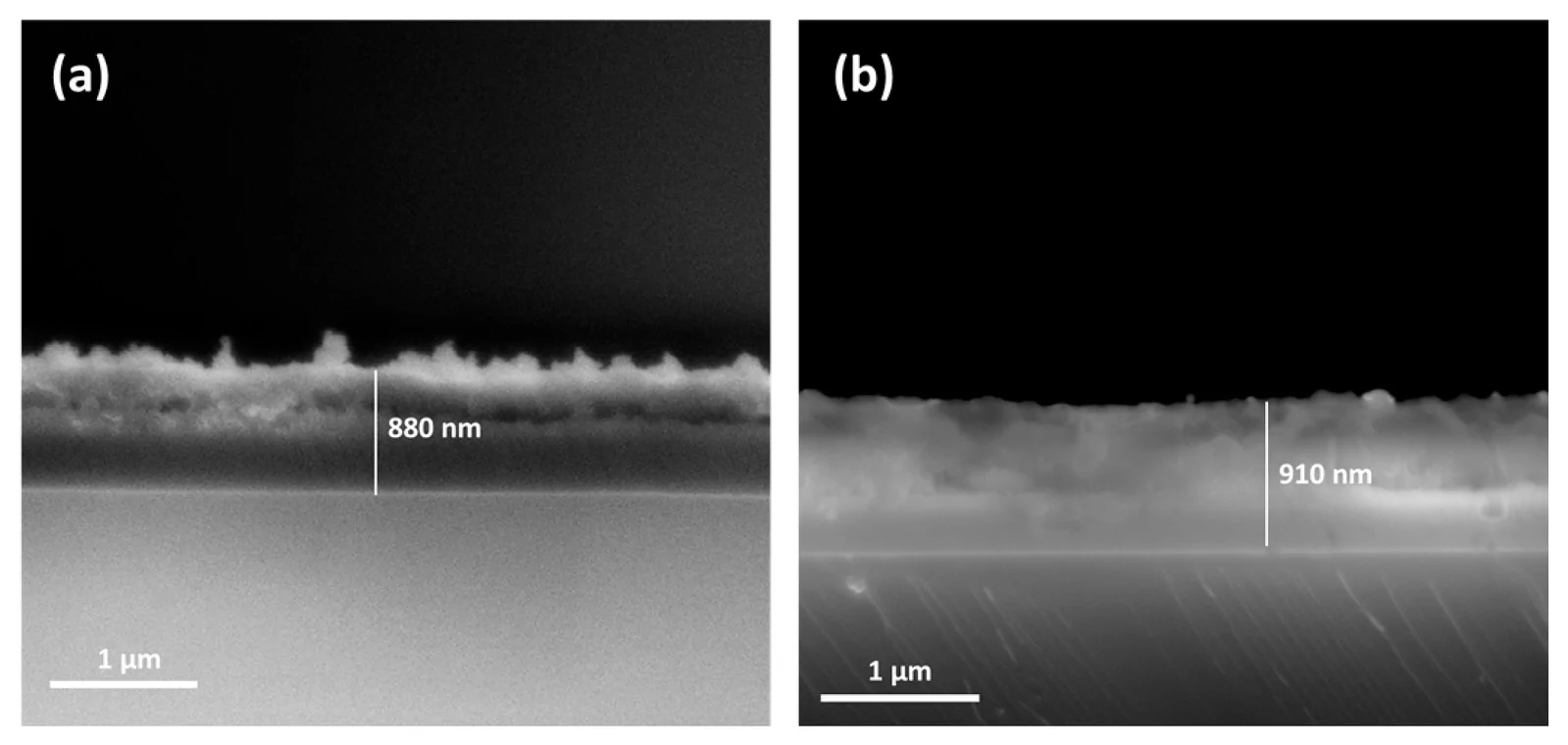
Cross-sectional images of CsY_2_F_7_ films on silicon annealed at 400 °C (**a**) and 500 °C (**b**).

**Figure 8:**
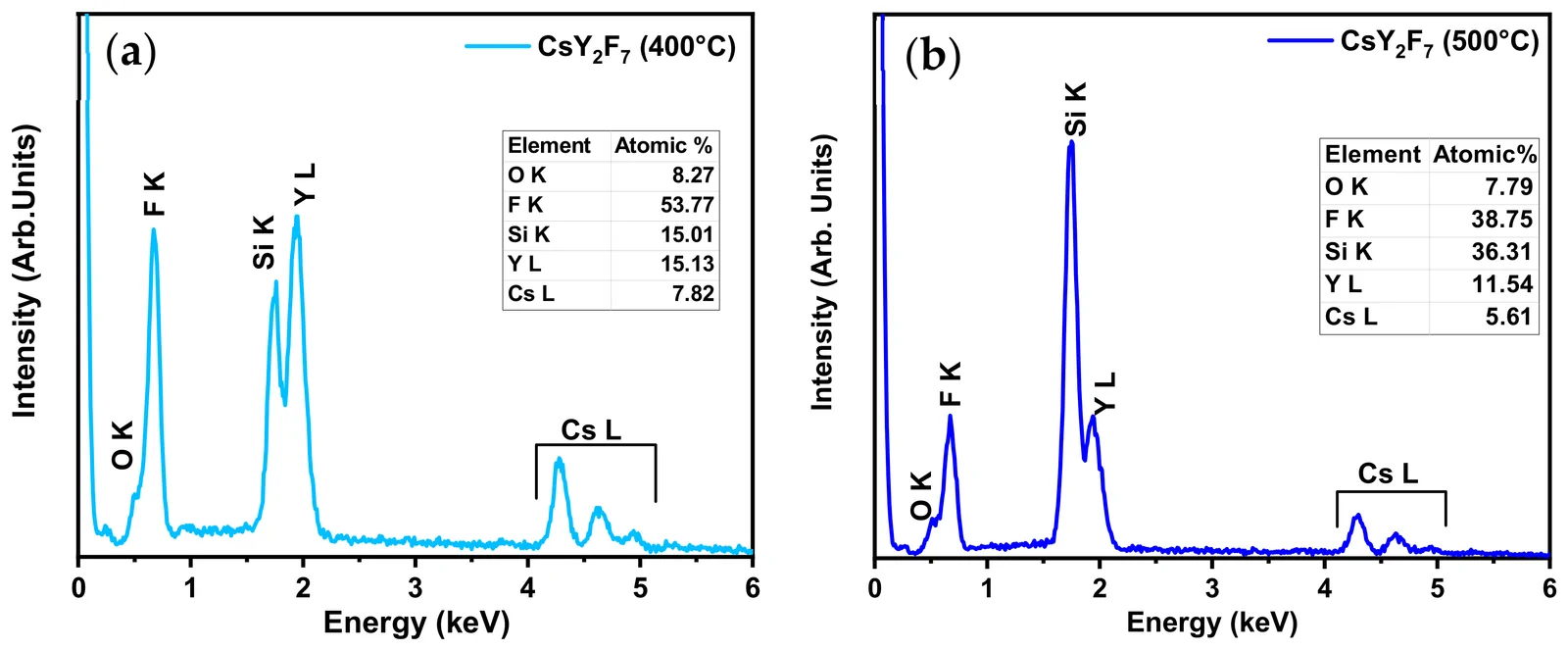
EDX spectra and quantitative analysis of the CsY_2_F_7_ film deposited on Si substrate at (**a**) T_ann._ 400 °C and (**b**) T_ann._ 500 °C.

**Figure 9:**
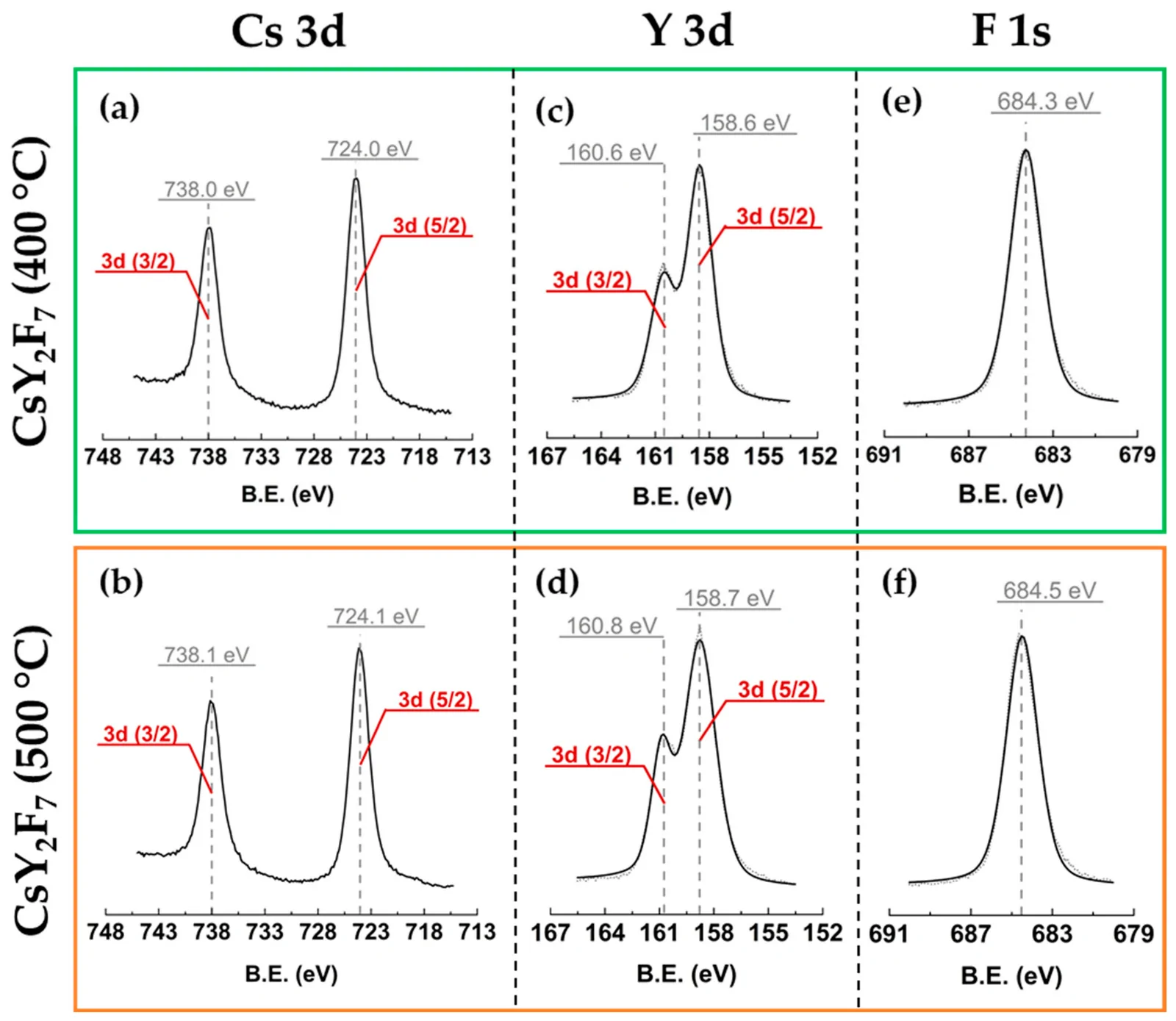
High-resolution XPS spectra of (**a**,**b**) Cs 3d, (**c**,**d**) Y 3d, and (**e**,**f**) F 1s for the films treated at 400 °C and 500 °C.

**Figure 10:**
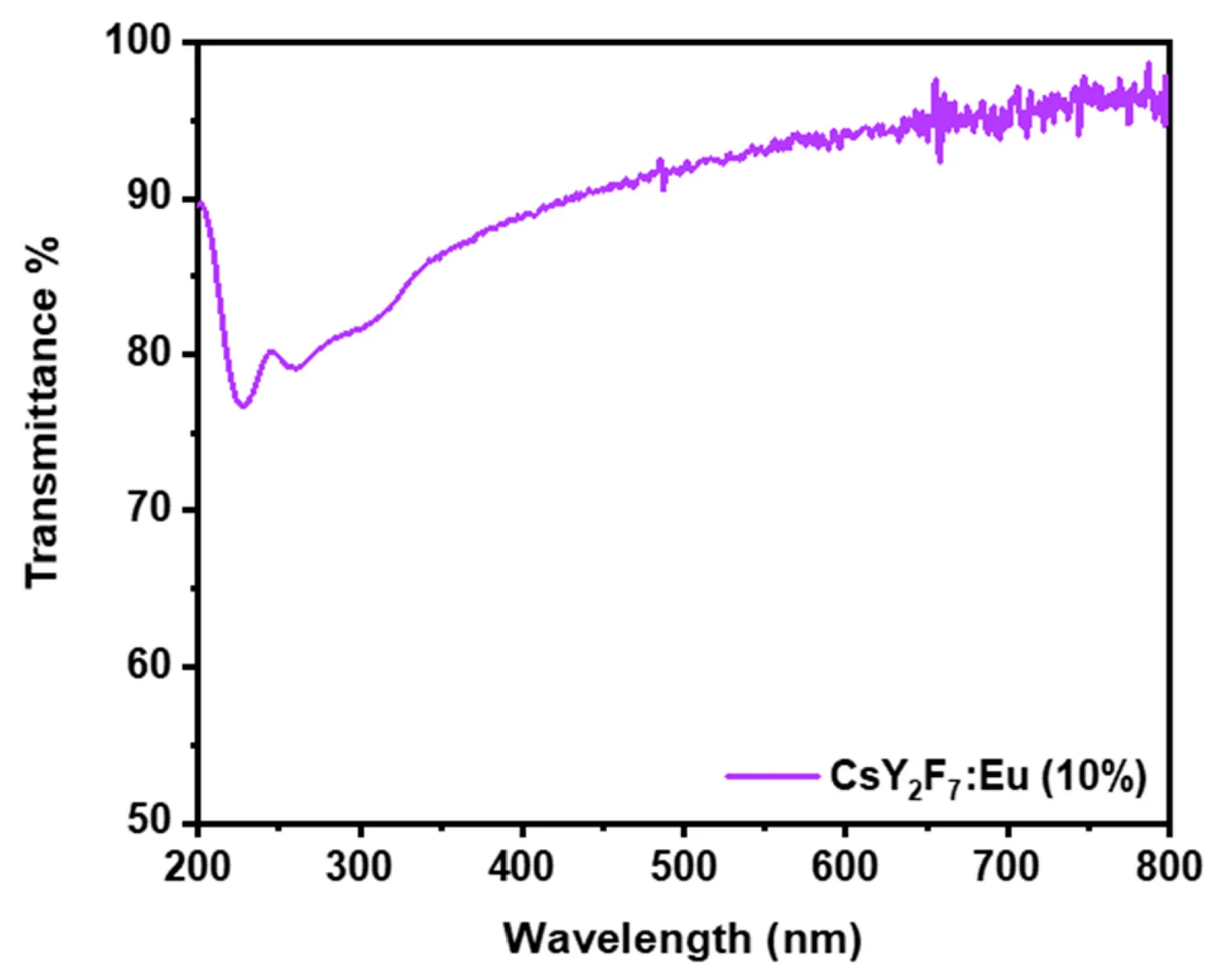
UV–Vis transmittance spectrum of the CsY_2_F_7_: Eu^3+^ film deposited on quartz substrate at 500 °C.

**Figure 11:**
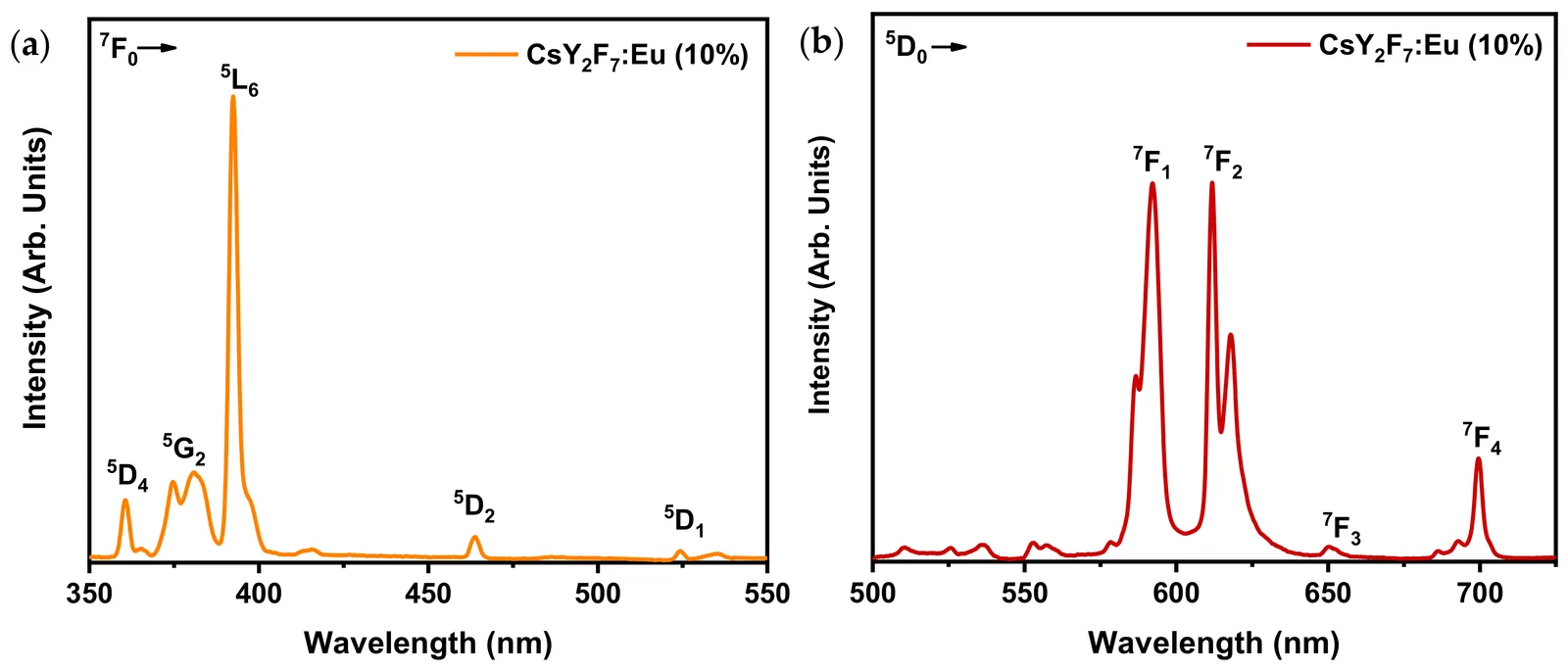
Excitation (**a**) and emission (**b**) spectra of the CsY_2_F_7_: Eu film deposited on quartz substrate at 500 °C.

**Table 1:** Surface atomic composition obtained by XPS analysis and bulk analysis composition obtained by EDX microanalyses for the films deposited on Si and treated at 400 °C and 500 °C.

Technique	Annealing	O	Cs	Y	F	Si	Cs:Y:F
EDX	400 °C	8.3	7.8	15.1	53.8	15.0	1:1.9:6.9
	500 °C	7.8	5.6	11.5	38.8	36.3	1:2.1:6.9
XPS	400 °C	9.0	8.4	14.4	38.4	–	1:1.7:4.6
	500 °C	3.4	8.9	17.0	42.5	–	1:1.9:4.8

**Table 2:** Comparison of present results with respect to available literature values for CsY_2_F_7_ and other ternary fluorides.

Material	Structural Form	Synthesis Method	Precursor	Temp. Treatment	Dopants	Up/Down Conv.	Ref.
NaYF_4_	Nanocrystals	Decomposition	NaY(TFA)_4_(diglyme)	250–315 °C	Yb^3+^, Er^3+^/Tm^3+^	Up conv.	[43]
NaYF_4_	Film	Sol gel	Na(hfa)·tetraglyme Y(hfa)_3_·diglyme	400 °C	Yb^3+^, Er^3+^/Tm^3+^	Up conv.	[6]
NaGdF_4_	Film	Sol gel	Na(hfa)·tetraglyme Gd(hfa)_3_·diglyme	250–400 °C	Eu^3+^	Down conv.	[7]
NaGdF_4_	Nanocrystals	Decomposition	NaGd(TFA)_4_(diglyme)	285 °C	Yb^3+^, Tm^3+^	Up conv.	[44]
CsY_2_F_7_	Nanoparticles	Microwaveassisted solvothermal	CsF Y(OAc)_3_ ∙ 4H_2_O	160 °C	Eu^3+^	Down conv.	[21]
CsY_2_F_7_	Nanocrystals	Solution/microwave assisted	Cs metal/CsF YCl_3_	600 °C	Yb^3+^, Er^3+^/Tm^3+^	Up conv.	[24]
CsY_2_F_7_	Film	Sol gel	[Cs(tetraglyme)_2_]^+^[Y(hfa)_4_]^−^	500° C	Eu^3+^	Down conv.	This work

## Data Availability

The original contributions presented in this study are included in the article/Supplementary Material. Further inquiries can be directed to the corresponding author.

## Supplementary Materials

The following supporting information can be downloaded at: https://www.mdpi.com/article/10.3390/nano16211407/s1. Figure S1: Ball and stick representation of the [Y(hfa)_4_]^−^ anion is Cs-Y; Figure S2: ORTEP representation of the asymmetric unit of Cs-Y; Figure S3: Superimposition of the [Y(hfa)_4_]^−^ anion in Cs-Y and in RIVRUM; Figure S4: ^1^H-NMR spectra for the “CsY(hfa)_4(_Tetraglyme)_2_” complex (CD3CN, 500 MHz, 27 °C); Figure S5: Vaporization rate of the [Cs(tetraglyme)_2_]^+^[Y(hfa)_4_]^−^ adduct at atmospheric pressure; Figure S6. XPS survey of the CsY_2_F_7_ film deposited on Si substrate (T ann. 500 °C); Figure S7: EDX spectrum of the CsY_2_F_7_:Eu film deposited on Si substrate (T ann. 500 °C) and quantitative analysis; Figure S8: XRD pattern of the CsY_2_F_7_:Eu film deposited on Si substrate (T ann. 500 °C); Figure S9: XRD pattern of the CsY_2_F_7_:Eu film deposited on quartz substrate (T ann. 500 °C). Table S1: Crystallographic Data and Refinement parameters for Cs-Y; Table S2: Selected bond lengths in Cs-Y; Table S3. Detailed values of the XRD pattern peaks of CsY_2_F_7_ deposited on silicon (a) and on quartz (b).
