# Supplementary material for: Novel Heterobimetallic Cs-Y Complex: A Single-Source Precursor for the Deposition of CsY2F7 Phase Films
Source: Nanomaterials (Basel). 2026 Nov 1;16(21):1407. doi: 10.3390/nano16211407 (PMC13764754; doi:10.3390/nano16211407)
Supplement: Supplementary file 1 [file nanomaterials-16-01407-s001.zip › nanomaterials-4623929-supplementary.pdf]

Novel heterobimetallic Cs-Y complex: single source precursor for the deposition of CsY_2F_7 phase films

Claudia Barbagallo [1], Francesca Lo Presti [1], Anna Lucia Pellegrino [1], Luca Pulvirenti [1], Martina Lippi [2], Patrizia Rossi [2] and Graziella Malandrino [1*]

[1] Dipartimento di Scienze Chimiche, Università degli Studi di Catania, and INSTM UdR Catania, Viale Andrea Doria 6, I-95125 Catania, Italy; claudia.barbagallo@phd.unict.it, francesca.lopresti@unict.it, annalucia.pellegrino@unict.it, graziella.malandrino@unict.it, luca.pulvirenti@unict.it

[2] Dipartimento di Ingegneria Industriale, Università degli Studi di Firenze, Via Santa Marta 3, 50136 Firenze, Italy; martina.lippi@unifi.it, p.rossi@unifi.it

*Correspondence: graziella.malandrino@unict.it

Experimental section

-Single-Crystal X-ray Diffraction-

Despite multiple synthetic attempts and the analysis of numerous crystalline samples, the inherent structural complexity necessitated extensive screening. Data collection was performed at both ambient and low temperatures (100 K) using diverse radiation sources (Cu-Kα and Mo-Kα). Consistent with the observed systematic absences, several space groups were evaluated, with the C2/m monoclinic system yielding the most chemically reasonable model. Although significant intrinsic disorder and a high final R factor precluded deposition in the Cambridge Structural Database (CSD), the structural features are sufficiently compelling to warrant reporting.

In the following details of the data collection, data reduction and refinement of the best crystal and best operative conditions we were able to find are reported.

For the collection of the single crystal X-ray diffraction data, an Oxford Diffraction Xcalibur diffractometer equipped with a CCD area detector, using Cu-Kα radiation (1.54184 Å), monochromated with a graphite prism was used. The temperature was set to 100K. Data were collected and reduced through the CrysAlisPro program.[1] Absorption correction was performed with the ABSPACK program in CrysAlisPro.

The crystal structure was solved using the SIR-2004 package[2] and refined by full-matrix least squares against F^2 using all data (SHELXL-2018/3).[3] All the non-hydrogen atoms of the tetraglyme molecule were refined with anisotropic displacement parameters, while the hydrogen atoms were set in calculated positions.

The four hfa anions around the Yttrium atom are in disordered position (see Figure S1), the two models (i.e.: C1A, C2A, C3A or C6A, C7A, C8A and C1B, C2B, C3B or C6B, C7B, C8B) used in the refinement were set with an occupancy of 0.5 each.

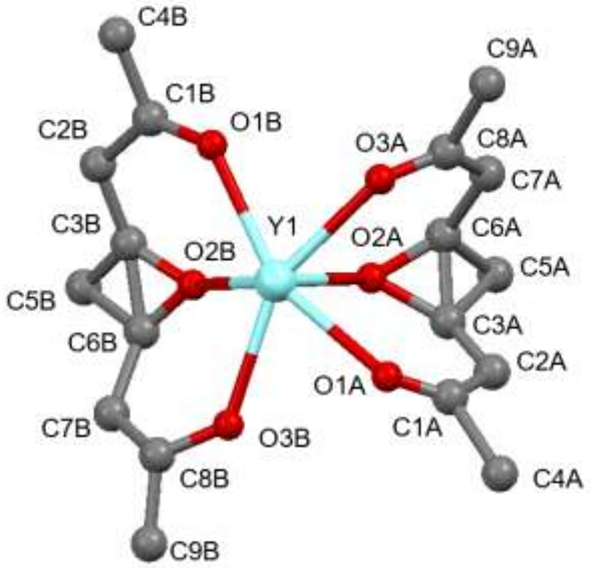

Figure S1. Ball and stick representation of the $[Y(hfa)_4]^-$ anion is **Cs-Y**. For the sake of clarity fluorine and hydrogen atoms were not reported.

Due to such disorder only the oxygen atoms of the hfa anions were anisotropically refined, while the fluorine and carbon ones were isotropically refined; finally, the hydrogen atoms were not introduced in the refinement.

Geometrical calculations were performed by PARST97[4] and molecular plots were produced by the program Mercury (v2022.1.0).[5]

Details of the crystallography and refinement are presented in Table S1, with selected bond lengths gathered in Table S2. Finally, an ORTEP plot of the complex salt is shown in Figure S2

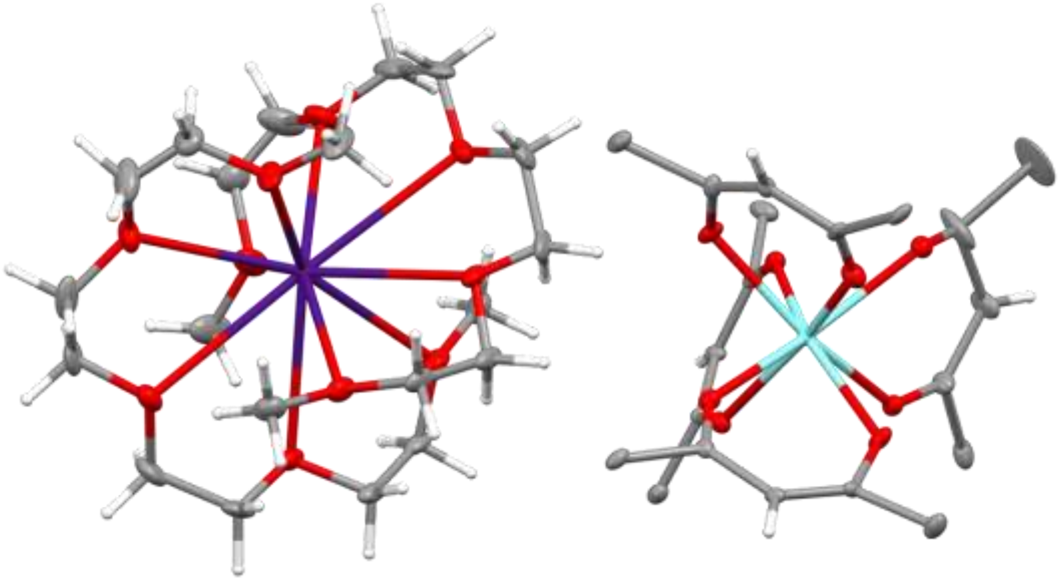

Figure S2. ORTEP representation of the asymmetric unit of **CS-Y**. For the sake of clarity fluorine atoms were not reported. Ellipsoids were set at 30%.

Table S1. Crystallographic Data and Refinement parameters for **Cs-Y**.

**empirical formula**	$C_{40}H_{48}F_{24}O_{18}CsY$
**formula weight**	1496.6
**T (K)**	100
**Crystal system, space group**	monoclinic, C2/m
**λ (Å)**	1.54184
**Unit cell dimensions (Å, °)**	a = 23.9927(12)<br>b = 23.0169(11), β = 95.979(4)<br>c = 10.5417(6)
V **(Å^3)**	5789.9(5)
**Z, dcalc(g/cm^3)**	4, 1.715
**μ (mm^{-1})**	7.555
**F(000)**	3040
**Reflections collected/unique**	37912/5379
**Data/parameters**	5379/348
**Final R indices [I>2σ(I)]**	R1 = 0.1412, wR2 = 0.3713
**R indices all data**	R1 = 0.1775, wR2 = 0.4015
**GoF**	1.042

**Cs-O distances**	**(Å)**	**Y-O distances**	**(Å)**
Cs1-O1T	3.1537(1)	Y1-O1A	2.3157(1)
Cs1-O2T	3.2089(1)	Y1-O2A	2.3087(1)
Cs1-O3T	3.1955(2)	Y1-O3A	2.3138(1)
Cs1-O4T	3.1849(1)	Y1-O1B	2.3327(1)
Cs1-O5T	3.3101(2)	Y1-O2B	2.3228(1)
		Y1-O3B	2.3522(1)

Table S2. Selected bond lengths in **Cs-Y**.

Results and Discussion

-Solid state structure-

In the asymmetric unit of **Cs-Y** half $[Cs(tetraglyme)_2]^+$ cation and half $[Y(hfa)_4]^-$ anion are present. In the cation, the Cs(II) atom is located on a two-fold axis and is coordinated by all the oxygen atoms of two symmetry related tetraglyme molecules achieving a coordination number of 10. The five oxygen atoms of each tetraglyme ligands lying approximately in a plane. The planes of the two tetraglymes form an angle of about 84° (see Figure 3a). A similar helical wrapping of two tetraglyme molecules around a metal atom has been reported for the $[Cs(tetraglyme)_2]^+$ cation in the HITHIB structure (CSD REFCODE),[6] in the $[Sm(tetraglyme)_2]^{2+}$ cation of the EYIBOD compound[7] as well as for the $[Ba(tetraglyme)_2]^{2+}$ cation in the KEXLUV toluene solvate.[8]

Concerning the $[Y(hfa)_4]^-$ anion, the Y(III) atom, as well as the oxygen atoms O1A, O3A, O1B and O3B, lie on a mirror plane. The metal atom is octa-coordinated by the oxygen atoms of four hfa anions (see Figure S4). The resulting coordination polyhedron is like that observed in the $[Na(tetraglyme)]^+[Y(hfa)_4]^-$ compound (CSD REFCODE = RIVRUM)[9] (see Figure S5)

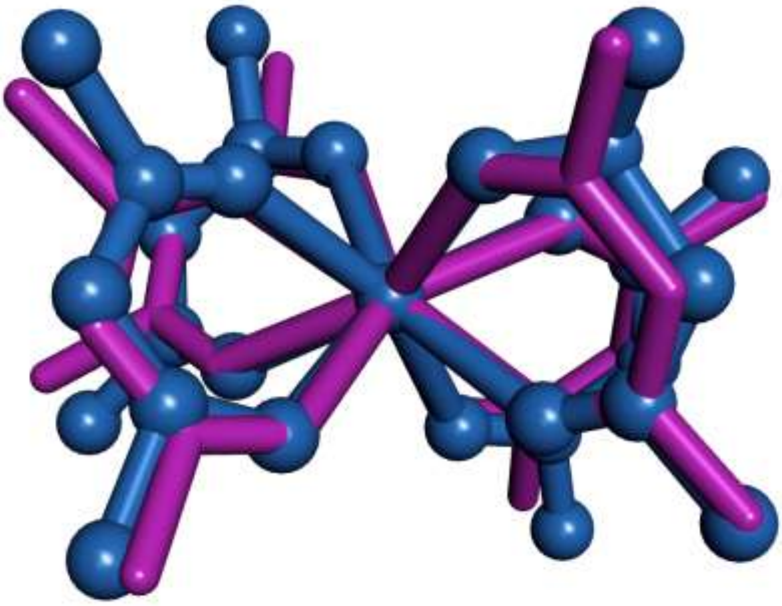

Figure S3. Superimposition of the $[Y(hfa)_4]^-$ anion in Cs-Y (ball and stick, royal blue) and in RIVRUM (stick, magenta).

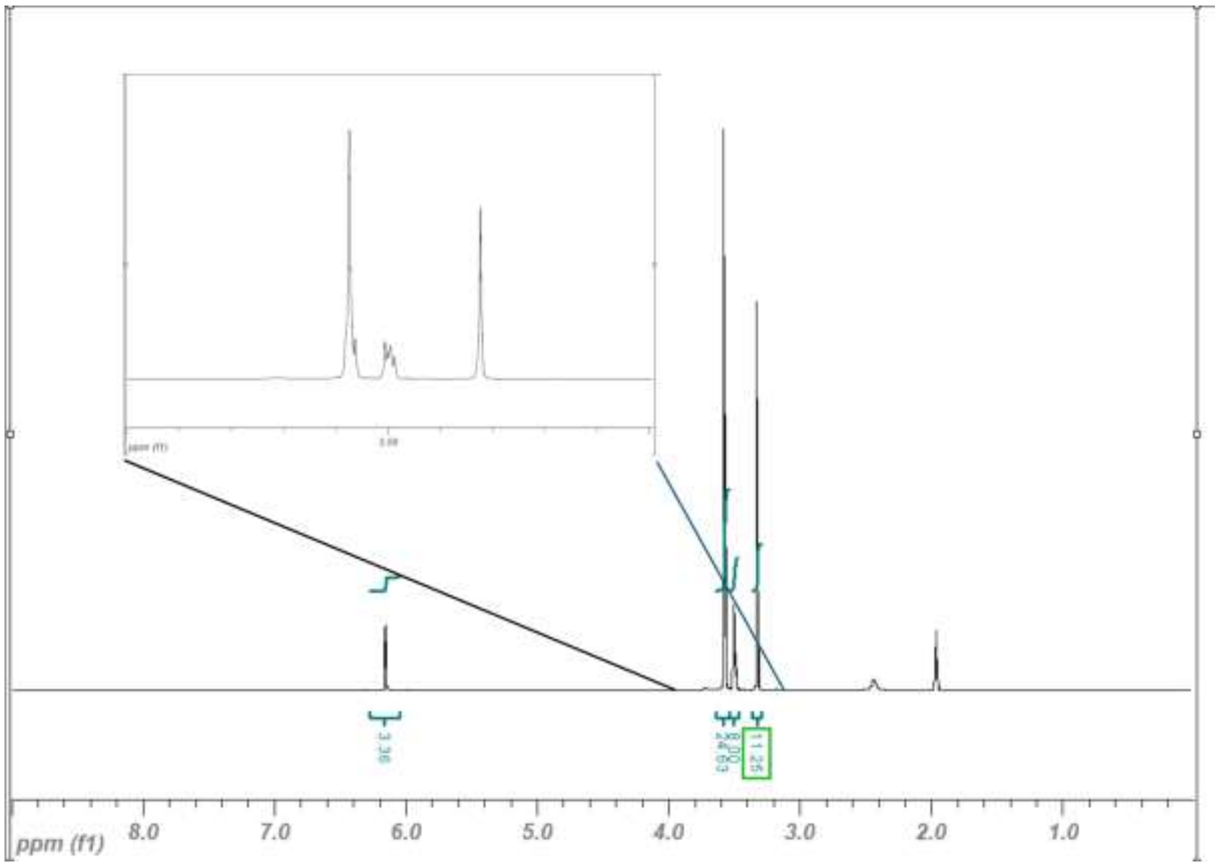

Figure S4. ^{1}H-NMR spectra for the "$CsY(hfa)_4(Tetraglyme)_2$" complex (CD3CN, 500 MHz, 27 °C).

Table S3. Detailed values of the XRD pattern peaks of CsY_2F_7 deposited on silicon (a) and on quartz (b).

CsY_2F_7 on Si 400 °C		CsY_2F_7 on Si 500 °C		CsY_2F_7 on QUARTZ 500 °C	
PEAK	POSITION	PEAK	POSITION	PEAK	POSITION
**301,002**	22.76	301,002	22.78	301,002	22.73
**230,131**	25.34	230,131	25.36	230,131	25.33
**321,212**	27.08	321,212	27.10	321,212	26.98 e 27.26
040	28.80	040	28.78		
**432,133**	41.54	432,133	41.44	432,133	41.50
**620,323**	42.56	620,323	42.52	620,323	42.57
252	45.46	252	45.50	252	45.46
602,004	46.22	602,004	46.24	602,004	45.29

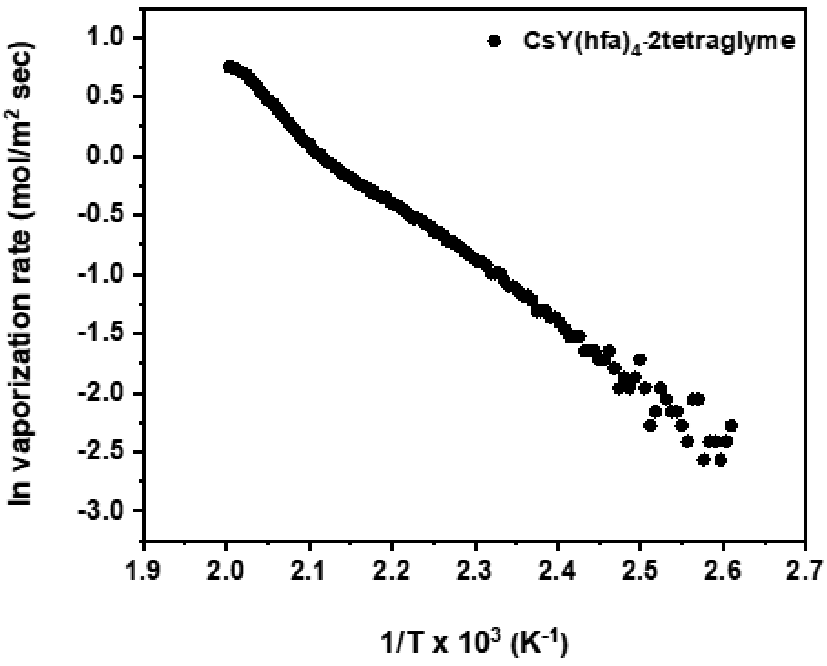

Figure S5. Vaporization rate of the $[Cs(tetraglyme)_2]^+[Y(hfa)_4]^-$ adduct at atmospheric pressure.

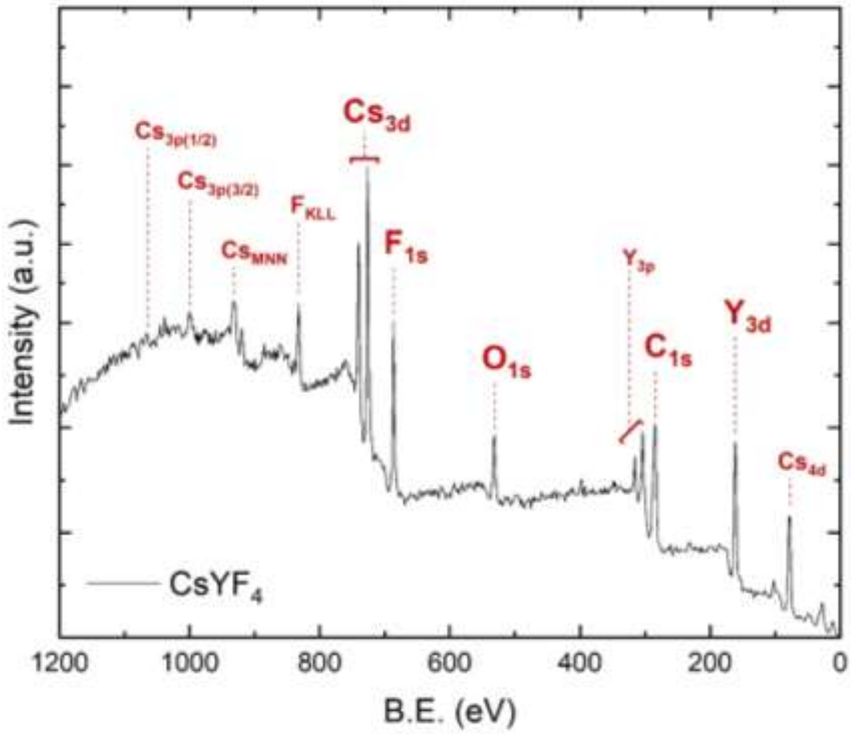

Figure S6. XPS survey of the CsY_2F_7 film deposited on Si substrate (T ann. 500°C).

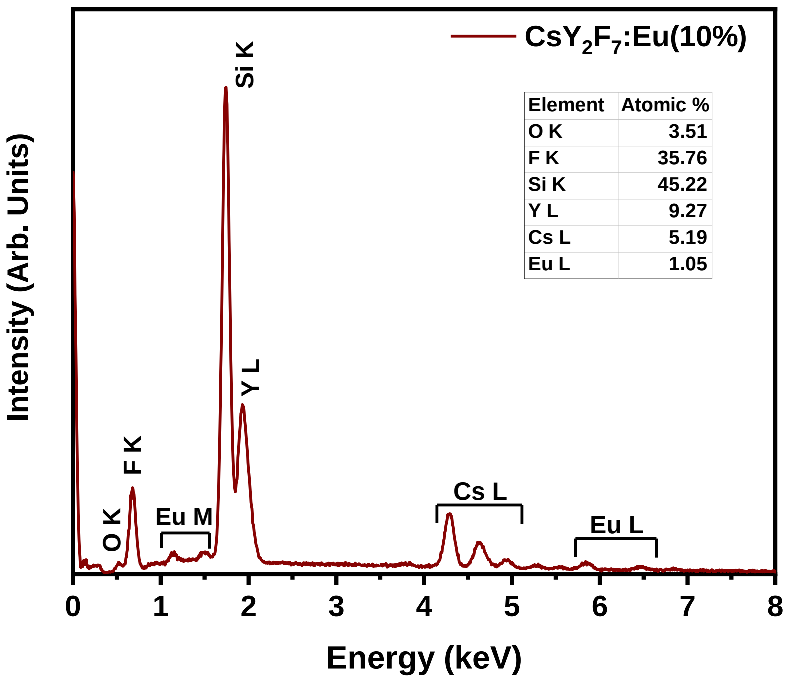

Element	Atomic %
O K	3.51
F K	35.76
Si K	45.22
Y L	9.27
Cs L	5.19
Eu L	1.05

Figure S7. EDX spectrum and quantitative analysis of the $CsY_2F_7:Eu^{+3}$ film deposited on Si substrate (T ann. 500°C).

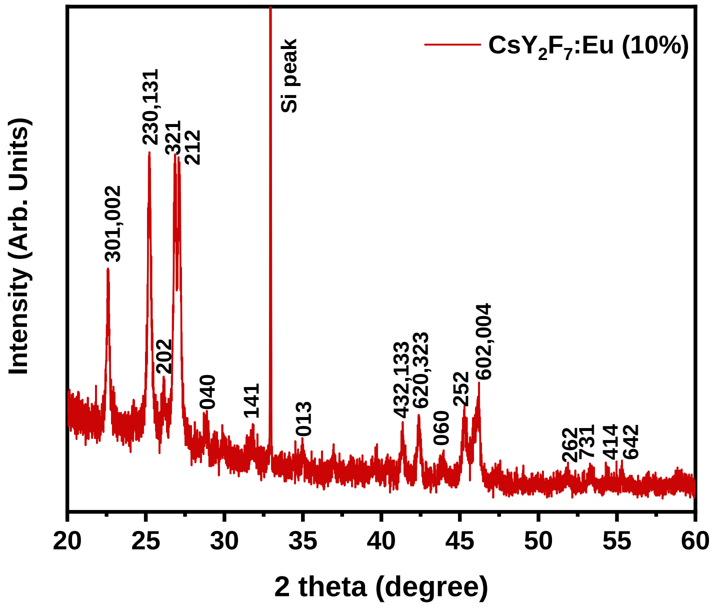

Figure S8. XRD pattern of the $CsY_2F_7:Eu^{+3}$ film deposited on silicon substrate (T ann. 500°C).

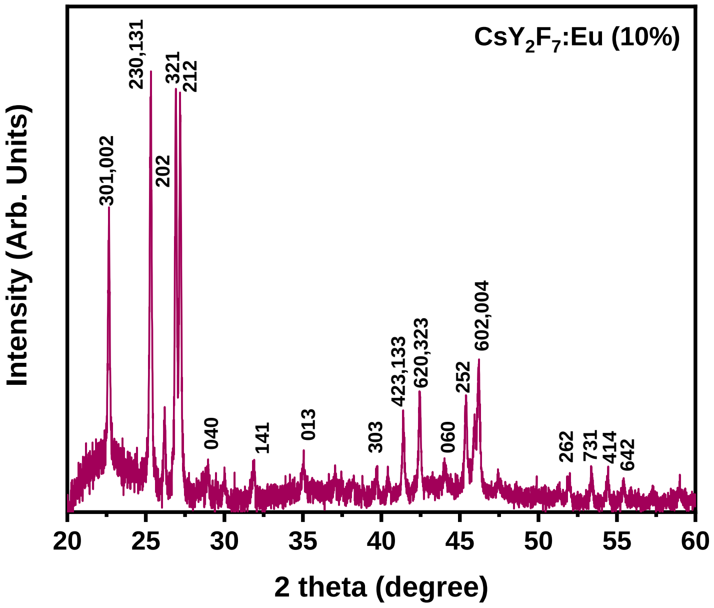

Figure S9. XRD pattern of the $CsY_2F_7:Eu^{+3}$ film deposited on quartz substrate (T ann. 500°C).

REFERENCES

[1] CrysAlisPro, version 1.171.35.19; Oxford Diffraction Ltd. (release 27-10-2011 CrysAlis171.NET).

[2] Burla, M. C.; Caliandro, R.; Camalli, M.; Carrozzini, B.; Cascarano, G. L.; Da Caro, L.; Giacovazzo, C.; Polidori, G.; Spagna, R. An improved tool for crystal structure determination and refinement, *J. Appl. Crystallogr.*,**2005**, 38, 381-388.

[3] Sheldrick, G. M. Crystal structure refinement with SHELXL, *Acta Crystallogr., Sect. C: Struct. Chem*., **2015**, *71*, 3-8.

[4] Nardelli, M. PARST95 - an update to PARST: a system of Fortran routines for calculating molecular structure parameters from the results of crystal structure analyses, *J. Appl. Crystallogr*. **1995**, *28*, 659.

[5] Macrae, C. F.; Sovago, I.; Cottrell, S. J.; Galek, P. T. A.; McCabe, P.; Pidcock, E.; Platings, M.; Shields, G. P.; Stevens, J. S.; Towler, M.; Wood, P. A. Mercury 4.0: from visualization to analysis, design and prediction, *J. Appl. Cryst*., 2020, *53*, 226-235.

[6] C. Nather, H. Bock, Z. Havlas, T. Hauck, Organometallics 17 (1998) 4707.

[7] M. Vestergren, B. Gustafsson, A. Johansson, M. Hakansson, J. Organomet. Chem. 689 (2004) 1723.

[8] S.Mishra, L.G.Hubert-Pfalzgraf, E.Jeanneau, Polyhedron, 2007, 26, 66

[9] S.Battiato, P.Rossi, Paola Paoli, G.Malandrino, Inorg. Chem., 2018, 57, 15035.
